# National Rare Diseases Registry System (NRDRS): China’s first nation-wide rare diseases demographic analyses

**DOI:** 10.1186/s13023-021-02130-7

**Published:** 2021-12-18

**Authors:** Jian Guo, Peng Liu, Limeng Chen, Haohan Lv, Jie Li, Weichao Yu, Kaifeng Xu, Yicheng Zhu, Zhihong Wu, Zhuang Tian, Ye Jin, Rachel Yang, Weihong Gu, Shuyang Zhang, the Administrative Group of National Rare Diseases Registry System of China

**Affiliations:** 1 The Administrative Group of National Rare Diseases Registry System of China, Beijing, 100730 China; 2 Department of Cardiology, State Key Laboratory of Complex Severe and Rare Diseases, Peking Union Medical College Hospital, Chinese Academy of Medical Sciences and Peking Union Medical College, Beijing, 100730 China; 3 Medical Research Center, State Key Laboratory of Complex Severe and Rare Diseases, Peking Union Medical College Hospital, Chinese Academy of Medical Sciences and Peking Union Medical College, Beijing, 100730 China; 4 Department of Nephrology, State Key Laboratory of Complex Severe and Rare Diseases, Peking Union Medical College Hospital, Chinese Academy of Medical Sciences and Peking Union Medical College, Beijing, 100730 China; 5 Department of Respiratory and Critical Care Medicine, State Key Laboratory of Complex Severe and Rare Diseases, Peking Union Medical College Hospital, Chinese Academy of Medical Sciences and Peking Union Medical College, Beijing, 100730 China; 6 Department of Neurology, State Key Laboratory of Complex Severe and Rare Diseases, Peking Union Medical College Hospital, Chinese Academy of Medical Sciences and Peking Union Medical College, Beijing, 100730 China; 7 China Alliance for Rare Diseases (CHARD), Beijing, China

**Keywords:** China, Rare diseases, Registry, Database

## Abstract

**Background:**

China has made tremendous progresses in serving the needs of its people living with rare diseases in the past decade, especially over the last 5 years. The Chinese government’s systematic approach included a series of coordinated initiatives, amongst these are: forming the Rare Disease Expert Committee (2016), funding the “Rare Diseases Cohort Study” (2016–2020), and publishing its first “Rare Disease Catalog” (2018). Herein, we present the National Rare Diseases Registry System (NRDRS)—China’s first national rare diseases registry, and the analysis of cases registered in the first 5 years ending Dec 31, 2020.

**Results:**

The total 62,590 cases covered 166 disease/disease types, forming 183 disease cohorts. The data from nearly 22% of them (13,947 cases) is also linked to valuable biological samples. The average age of definitive diagnosis was 30.88 years; 36.07% of cases were under 18 years of age. Regional distribution analysis showed 60% of cases were from the more developed, wealthier East and North China, suggesting the local availability of quality care and patients’ financial status were key access factors. Finally, 82.04% of cases were registered from the five clinical departments: Neurology, Endocrine, Hematology, Cardiovascular, and Nephrology, suggesting that either these are most affected by rare diseases, or that there were disease non-specific ascertainment factors.

**Conclusions:**

The preliminary analysis of the first 5-year’s data provides unique and valuable insight on rare disease distribution in China, and higlights the directions for enhancing equity, scale and utility.

**Supplementary Information:**

The online version contains supplementary material available at 10.1186/s13023-021-02130-7.

## Introduction

The lack of common definition of rare disease makes it challenging to calculate the total number and types of rare diseases in the world. It’s frequently cited there are over 7000 rare diseases in total [1], although recently Haendel et al. gave an estimate of over 10,000 [2]. Rare disease definition is not just a scientific matter, it’s also heavily influenced by the social economic status of a country such that high income countries include diseases with much higher (0.04–0.06%) prevalence than low and middle income countries. In the US, a rare disease or condition is one affects less than 200,000 people; in Europe, it affects less than 1 in 2000; and in Japan, less than 1 in 2500 [3]. China doesn’t yet have a nationally endorsed rare disease definition [4]. Using Orphanet’s evidence-based estimated population prevalence of rare diseases of 3.5–5.9% [5], we calculated China, with its population of 1.4 billion, has approximately 49–82 million Chinese suffering from a rare disease. Irrespective of the exact number, the societal burden is huge [6]. Despite of the staggering numbers of Chinese affected and the cumulative burden, rare diseases research has historically been limited to single disease or disease type and in tertiary hospitals only [7]. Knowledge sharing and collaborations among clinicians and hospitals were challenged and limited and there was a severe shortage of nationally coordinated research [8].

Rare diseases have gained increasing recognition as a public health priority in China [7, 9, 10]. Starting 2016, the Chinese government began to further tackle rare diseases with a more systematic, cohesive and integrated cross-sector approach. The Government formed the nation’s First Rare Diseases Expert Committee in Jan 2016 [11]. The multi-disciplinary committee served as a consultation and advisory committee to assist government on rare disease-related policy-making, funding, and other initiatives. In May 2018, China’s first “Rare Diseases Catalog” was jointly published by National Health Commission, Ministry of Science and Technology, Ministry of Industry and Information Technology, National Medical Products Administration, and National Administration of Traditional Chinese Medicine [12]. This was a major milestone for rare disease development in China. The first catalog listed 121 rare diseases and rare disease types. The Second Rare Diseases Expert Committee was formed in September 2020 [13], and is working on including more rare diseases into the catalog.

In 2016, Peking Union Medical College Hospital (PUMCH) was selected as the lead institution to build the first national rare disease registry (National Rare Diseases Registry System of China; NRDRS), as part of the “Rare Diseases Cohort Study” funded under the nation’s “13^th^ Five-Year Plan” (2016–2020), in the “Key Research & Development Program – Precision Medicine Initiative” [14]. Table 1 listed the twenty hospitals and universities granted under the “Rare Diseases Cohort Study” and the study’s 5 sub-areas.

**Table 1:** “Rare Disease Cohort Study” research project and participating institutions

Project areas	Participating hospitals
National Rare Diseases Registry System of China (NRDRS)	1. Chinese Academy of Medical Sciences, Peking Union Medical College Hospital
Rare Cardiac, Respiratory and Kidney Diseases	1. Chinese Academy of Medical Sciences, Beijing Fuwai Hospital* 2. Chinese PLA General Hospital 3. Shanghai Changzheng Hospital 4. The First Affiliated Hospital of Guangzhou Medical University
Rare Endocrine Metabolic and Hematological Diseases	1. Ruijin Hospital, Shanghai Jiaotong University, School of Medicine* 2. The First Affiliated Hospital, Zhejiang University, School of Medicine 3. Chinese Academy of Medical Sciences, Institute of Hematology and Blood Diseases Hospital 4. Shandong Provincial Hospital 5. Shandong Academy of Medical Sciences
Rare Neurological, Skeletal and Cutaneous Diseases	1. West China Hospital, Sichuan University* 2. Fudan University 3. Xiangya Hospital, Central South University 4. The First Hospital of China Medical University 5. Wenzhou Medical University
Rare Pediatric Diseases	1. Peking University First Hospital* 2. Capital Institute of Pediatrics 3. Xinhua Hospital Affiliated to Shanghai Jiao Tong University, School of Medicine 4. Beijing Children’s Hospital, Capital Medical University 5. China-Japan Friendship Hospital

^*^The lead institution in each project area

The National Rare Diseases Registry System of China, built as a critical part of the nation’s rare disease infrastructure, was an online rare diseases registry. The goal was to establish national rare disease registration standards, and to collect standardized rare disease data on cases nation-wide. The NRDRS also served to unify nation’s top rare disease centers of excellence to form a national collaborative network [14]. Herein, we present the methods employed for case registration, and the results of the demographic data analysis for the first 5 years, ending 31 Dec, 2020.

## Methods

### Principal Investigators (PIs) and participating hospitals

By December 2020, 231 registered users from nation’s 85 tertiary hospitals participated in the study. Among them, 81 were Principal Investigators, who were authorized to design new disease forms and start a new cohort for a specific disease/disease type. The remaining users (researchers and research assistants) were limited to using existing disease forms designed by PIs, and enter cases to the PIs’ existing cohorts. All researchers access was limited to only their own cases.

### Case selection and data entry

The National Rare Diseases Registry System only accepted cases that have a definitive diagnoses by Principal Investigators (PIs). The diagnosed disease must meet one of the following four criteria: 1, Must have an ORPHAcode. 2, Must have an OMIM code. 3, Must be included in China’s first Rare Disease Catalog. 4, Must be recorded in a published academic research paper. Case data were collected by the PIs or researchers, and registered in NRDRS by PIs, researchers or research assistants (RAs).

Two types of case forms were created for case registration: the General Form and the Individual Disease Form. The General Form contained basic information about all patients, including patient demographic, social-economic data, diagnostic evidence, biospecimen, genetic test results, and survival status (Additional file 1). Information on the General Form must be completed before a researcher could move on to the next stage: to enter data on the Individual Disease Forms. Data on Individual Diseases Forms were grouped into 8 categories: Basic information; Disease History; Physical Examination; Ancillary Examination; Therapeutic Intervention; Assessment Scale; Mixed (data belong to more than one category); and Other (data belong to none of the categories). There were 183 different Case Forms in the system, each designed by a lead expert for a particular disease and used by other researchers in the same cohort. This allowed disease-specific data elements to be captured in NRDRS while maintaining data standards across different researchers and hospitals. The initial participation of the project was limited to RD specialists from the 20 hospitals (Table 1). Clinical data forms were by and large designed by PIs themselves. Going forward, the Administrative Group of NRDRS plans to establish a NRDRS Academic Committee consisting of experts from different clinical departments, basic medical research team, and epidemologists. The Committee will periodically review and revise the cases forms and constantly improve the data elements. NRDRS allowed researchers to complete a case report in multiple steps. Only when all the required information was entered, did the status of the case record become “Completed”. Only completed cases were included in the study.

Follow-up on survival status for the past registered cases was undertaken by the PIs and their teams. The Administrative Group of NRDRS also assisted PIs in follow-up activities. For the new cases, the current improved data acquisition process will help reinforce the patient follow-up going forward.

## Results

As of December 2020, a total of 62,590 rare disease cases were registered in NRDRS, the majority of the cases were retrospectively ascertained by individual clinicians prior to the initiation of the NRDRS project. As a result, not all desired information was available for all patients. It should also be noted that due to various reasons, the only age information available to analyze was patient’s age at time of visiting the PI when a definitive diagnosis was made.

Our demographic analysis is shown in Table 2. The average age of obtaining a definitive diagnosis was 30.88 years; 36.07% under 18 years of age, 15.22% in age group 19–30, 13.93% in 31–40, 13.84% in 41–50, and 10.77% in 51–60, 7.23% in 61–70 and 2.94% in ages greater than 71 years of age. More male (55.92%) than female (44.08%) cases were registered in NRDRS.

**Table 2:** The distribution of gender and age in registered cases

Categories		Number	Percentage (%)
Gender	Male	34,857	55.92
	Female	27,475	44.08
Age (years)	0–18	18,311	36.07
	19–30	7,725	15.22
	31–40	7,070	13.93
	41–50	7,026	13.84
	51–60	5,466	10.77
	61–70	3,672	7.23
	≥ 71	1,492	2.94

**Fig. 1:**
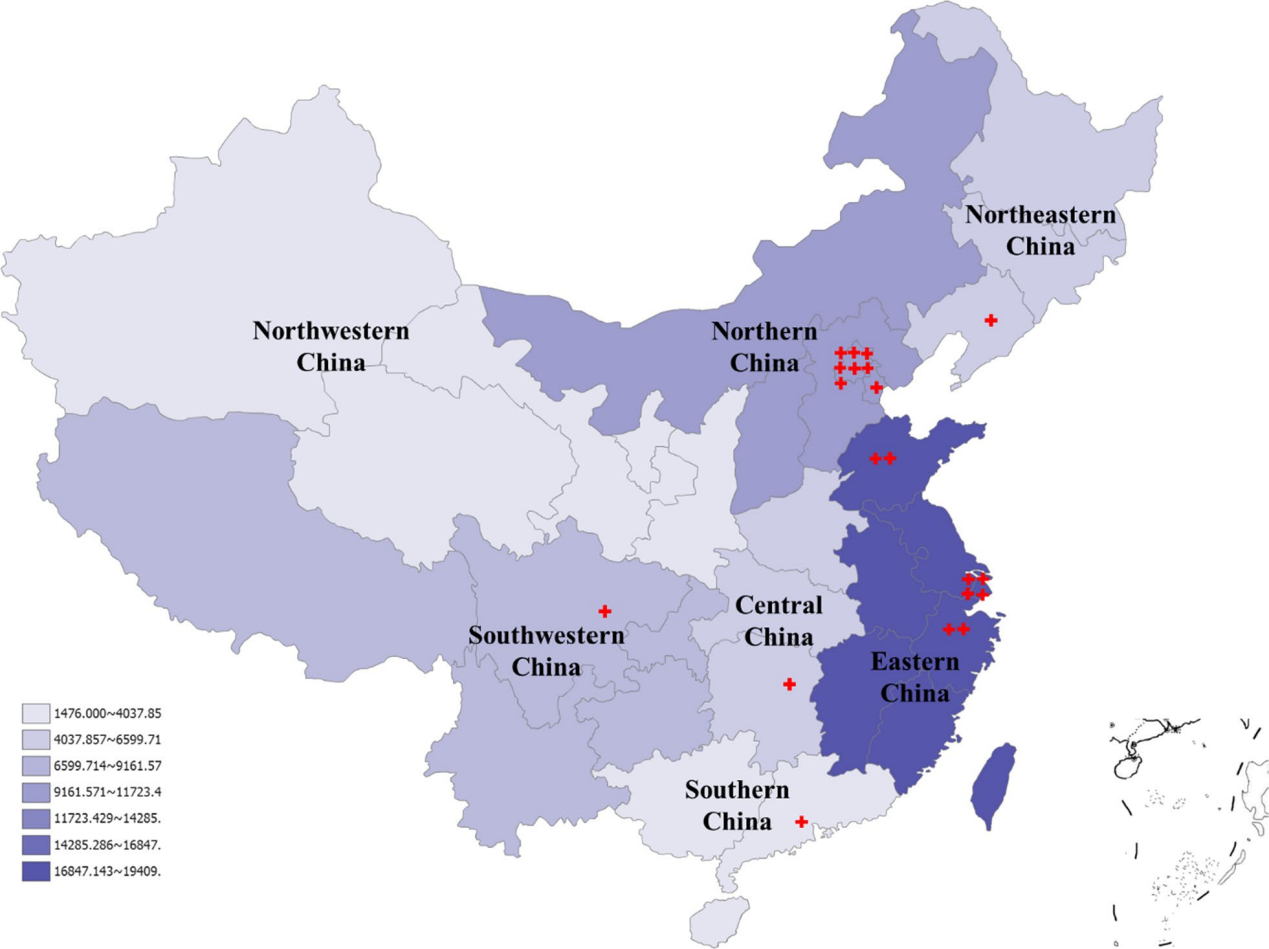
Regional distribution of registered cases. China’s Seven Regions and its Composition: North China: Beijing, Tianjin, Hebei, Shanxi and Inner Mongolia. Northeast China: Heilongjiang, Jilin and Liaoning. East China: Shanghai, Jiangsu, Zhejiang, Anhui, Jiangxi, Shandong, Fujian and Taiwan. South China: Guangdong, Guangxi, Hainan, Hong Kong and Macau. Central China: Henan, Hubei and Hunan. Southwest China: Chongqing, Sichuan, Guizhou, Yunnan and Tibet. Northwestern China: Shaanxi, Gansu, Qinghai, Ningxia and Xinjiang.  The twenty hospitals and universities funded under the “Rare Diseases Cohort Study

To assess the regional distribution of cases (Fig. 1), we used patient’s permanent residence instead of the temporary address, because a substantial proportion of China’s population is itinerant. China is divided into 7 regions: North China, Northeast China, East China, South China, Central China, Southwest China, and Northwest China. Each region includes several Chinese provinces, which are in close proximity and share certain geographical and cultural features. 60.91% of cases came from higher socioeconomic regions; East and North China. These two regions have the most abundant healthcare resources in China. People in these coastal provinces tend to be more informed, wealthier, and more likely to seek diagnosis and care. The high concentration of PIs and researchers in these two regions could also be a contributing factor.

**Fig. 2:**
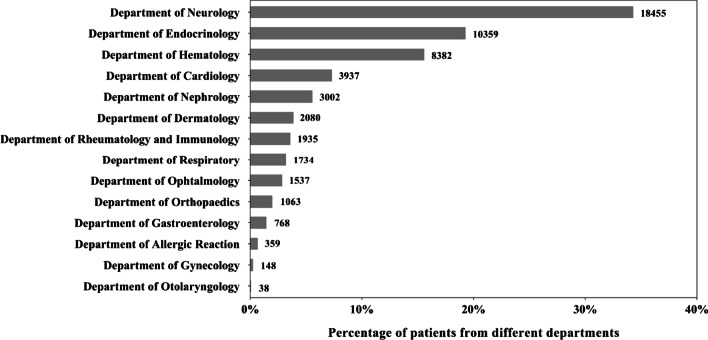
Distribution of rare disease cases across different clinical departments. *The patients registered from department of pediatrics (8793) were not included in this figure

As rare diseases often affect more than one body system, we wanted to look at case distribution within and across clinical specialty departments (Fig. 2). Rare diseases often impact multiple systems or organs, with compounding symptoms. Patients often went through multiple departments or even hospitals before achieving a definitive diagnosis. All cases in NRDRS were registered by the PIs and researchers who made the final diagnoses. We used the clinical department of PIs and researchers as the indicator of the system most affected by a rare disease.

Our analysis showed that 82.04% of the cases were registered from the following 5 clinical departments: Neurology, Endocrine, Hematology, Cardiovascular, and Nephrology (Fig. 2). We did not include the cases registered from the Pediatric Department in the General Hospitals, nor those from the Children’s hospitals, because cases registered from either places provided insufficient information on systems most impacted by the disease.

## Discussion

China is accelerating its advances to rare diseases through supporting coordinated researches with increased depth, breadth, inclusivity and equity. It is addressing historical limitation that has typicall focused on single disease/disease type, or within specific hospitals, and not enough attention was on setting data standards and quality. The National Rare Diseases Registry System is China’s first national rare disease registry that traverses multiple diseases and disease types. As of the end of 2020, the 62,590 registered cases covered 166 single rare disease /disease types, formed a total of 183 cohorts (17 diseases were represented by two cohorts). Furthermore, 13,947 cases contained valuable biological samples. See Additional file 2 for the list of cohorts and cases registered.

Approximately one third (61/183) of cohorts had currently more than 200 ascertained cases, contributing more than 92.11% of the total cases. Collectively they covered 52 rare diseases/disease types. Notably, 37 out of the 52 were already on the first “Rare Diseases Catalog”. At current level, the total number of cases for each disease/disease type is still too low to have meaningful inference on disease prevalence. Furthermore, majority of the cases were registered by PIs from the 20 hospitals in Table 1, thus with sampling bias. Wider participation of PIs from hospitals throughout the whole country, especially from pediatric hospitals or departments, will alleviate the bias. As the “Rare Diseases Expert Committee” continues its work to include more diseases/disease types in the catalog, we hope NRDRS would become an important source of information on rare diseases in China that will enable experts and policy makers to make decisions based on available data. Acknowledging and addressing potential biases will be critical for making the most informed decisions.

The first 62,590 cases registered in the initial phase of the project (2016–2020) came largely from the PIs of the initial 20 hospitals (Table 1). These were predominantly existing cases accumulated by the PIs from their clinical practices spanning many years, and some inevitably with missing data. Most of the cases came from many other parts of China, after long and odious journeys, got diagnosed and returned to their homes without valid contact information. Follow-up was extremely challenging. Missing critical data elements on existing cases proved one of the big challenges in the initial phase of the project. As we continued to upgrade NRDRS through a combination of better process control for case registration and improved case report form design, we already saw steady improvements on overall case quality. Furthermore, as more existing PIs exhausted their personal case collections, they would start to register more new cases with more data elements. Cases without required data points would not be accepted by the system. As NRDRS moves on to the next phase (the second 5-year plan), cases registered will be predominantly new cases (prospective) with more complete information. We anticipate this prospective ascertainment to increase case quality. The value of NRDRS lies largely with the data it collects, and the insight gained from analyzing the data.

While more than 50% of the rare diseases affect children [15, 16]. Our analysis showed only 36.07% of the cases in NRDRS were under 18 at time of definitive diagnosis. Several factors contributed to the under representation of the pediatric cases. First, NRDRS currently only registered living cases; those who died prior to registration were excluded. Child-onset rare diseases have high mortality, and 30% of the patients don’t live to 15 years old [16]. Second, it takes average 6 years for a patient to obtain a final diagnosis [17] and as age was coded at the time of defintive diagnosis, paediatric onset cases may be reflected as “adult cases” in NRDRS. The geographic imbalance of medical resources within China, may futher compound the effect of this for rural and remote patients. Finally, under-representation of children’s hospitals might have also contributed to the age shift to the right. Among the initial 20 hospitals who received the project funding, only 2 were children’s hospitals. While some of the participating general hospitals also have pediatric departments, children’s hospitals are far more concentrated with pediatric cases. It should be noted that in China only children age 14 and under are considered pediatric. Only 14.05% of total cases were registered from Children’s hospitals or Pediatric Departments in General Hospitals. Wakap et al. analyzed the Orphanet database and found 69.9% of the rare diseases are exclusively pediatric onset [5]. Rare disease research in children is of particular importance as genomic medicines become more established, and early diagnosis & intervention often lead to market improvement on outcome and later, quality of life [15, 18]. Recruiting more investigators from nation’s children’s hospitals, better methods that enhance data asertainment from rural and remote regions will help register more pediatric cases and enhance the value of NRDRS in the future.

## Conclusions

This initial collection of cases in NRDRS were mostly historical (retrospective) data from clinician’s own case collections, some dated years back, and with their own case report design. Most patients were lost to follow up. Significant number of cases were missing data points, such as age, sex, disease onset, and survival status etc. As NRDRS moved to register more new (prospective) cases, and with better case report form design, we expect to see improvement on case quality. Recruitment open to more hospitals and investigators, especially those from children’s hospitals, would expand disease coverage of NRDRS, and provide a more comprehensive view on China’s rare disease distribution and population, and pave the way for researches on natural disease history and disease burdens. Insights gained from NRDRS will provide researchers, policy-makers much needed evidence for more informed decision making about interventions to improve the lives of Chinese people and families living with rare diseases.

## Supplementary Information

**Additional file 1. **The items of General Form of NRDRS.

**Additional file 2. **List of Cohorts in NRDRS with Disease/Disease Type and Case Number.

## Data Availability

The datasets used and/or analyzed during the current study are available from the Administrative Group of National Rare Diseases Registry System of China (NRDRS) on reasonable request.

## Abbreviations

NRDRS: National Rare Diseases Registry System of China
PUMCH: Peking Union Medical College Hospital
PIs: Principal Investigators
RAs: Research assistants
