# Supplementary material for: National Rare Diseases Registry System (NRDRS): China’s first nation-wide rare diseases demographic analyses
Source: Orphanet J Rare Dis. 2021 Dec 18;16:515. doi: 10.1186/s13023-021-02130-7 (PMC8684272; doi:10.1186/s13023-021-02130-7)
Supplement: Supplementary file 1 — Additional file 1. The items of General Form of NRDRS. [file 13023_2021_2130_MOESM1_ESM.docx]

Additional file 1: The items of General Form of NRDRS

| Categories | Variables |
| --- | --- |
| Patients’ demographic | Unique ID^*^ |
|  | Name^*^ |
|  | Date of information collection^*^ |
|  | Date/Age of disease onset^*^ |
|  | Sex^*^ |
|  | Date of birth/Age of first contact of patient with doctors^*^ |
|  | Patient contact^*^ |
|  | Place of residence^*^ |
|  | Nation |
|  | ID Card Number |
|  | Education level |
|  | Marital status |
|  | Occupation type |
|  | Occupational status |
| Social-economic information | Social-economic information |
| Diagnostic evidence | Diagnosis of rare disease^*^ |
|  | Evidence of diagnosis^*^ |
| Biospecimen | Date of biospecimen storage^*^ |
|  | Type of biospecimen^*^ |
|  | Volume of biospecimen^*^ |
|  | Name of Biobank where the biospecimen is stored up^*^ |
| Genetic test results | Genetic test results |
| Survival status | Live - dead condition^*^ |
|  | Activity of daily living^*^ |
|  | Current drug treatment^*^ |
|  | Date of death^*^ |
|  | Reason of death^*^ |

^*^ Required fields
