# Supplementary material for: National Rare Diseases Registry System (NRDRS): China’s first nation-wide rare diseases demographic analyses
Source: Orphanet J Rare Dis. 2021 Dec 18;16:515. doi: 10.1186/s13023-021-02130-7 (PMC8684272; doi:10.1186/s13023-021-02130-7)
Supplement: Supplementary file 2 — Additional file 2. List of Cohorts in NRDRS with Disease/Disease Type and Case Number. [file 13023_2021_2130_MOESM2_ESM.docx]

Additional file 2: List of Cohorts in NRDRS with Disease/Disease Type and Case Number

| **Name of disease in NRDRS** | **Case Number** | **Name of Disease in Orphanet (ORPHAcode)** |
| --- | --- | --- |
| Hemophilia | 6187 | Hemophilia (448) |
| Idiopathic pulmonary arterial hypertension | 3121 | Idiopathic pulmonary arterial hypertension (275766) |
| Duchenne and Becker muscular dystrophy | 2622 | Duchenne and Becker muscular dystrophy (262) |
| Pituitary adenoma | 2493 | Pituitary adenoma (99408) |
| Spinocerebellar ataxia | 2481 | Spinocerebellar ataxia Type1 (98755), Spinocerebellar ataxia Type2 (98756), Spinocerebellar ataxia Type3 (98757), Spinocerebellar ataxia Type6 (98758), Spinocerebellar ataxia Type7 (94147), etc. |
| Autosomal dominant polycystic kidney disease | 2270 | Autosomal dominant polycystic kidney disease (730) |
| Alport syndrome | 2111 | Alport syndrome (63) |
| Myasthenia gravis | 2096 | Myasthenia gravis (589) |
| Primary Dystonia | 2081 | Primary dystonia DYT13 type (98807), Primary dystonia DYT17 type (370103), Primary dystonia DYT2 type (99657), Primary dystonia DYT21 type (306734), Primary dystonia DYT27 type (464440), Primary dystonia DYT4 type (98805), Primary dystonia DYT6 type (98806) |
| Congenital muscular dystrophy | 2071 | Congenital muscular dystrophy (97242) |
| Phenylketonuria | 1849 | Phenylketonuria (716) |
| Cushing syndrome | 1805 | Cushing syndrome (553) |
| Congenital adrenal hyperplasia | 1748 | Congenital adrenal hyperplasia (418) |
| Methylmalonic acidemia | 1675 | Methylmalonic acidemia due to methylmalonyl-CoA epimerase deficiency (308425), Methylmalonic aciduria due to transcobalamin receptor defect (280183) |
| Genetic malformation syndrome with short stature | 1650 | Genetic malformation syndrome with short stature (183570) |
| Multiple sclerosis | 1504 | Multiple sclerosis variant (228145) |
| Congenital hypogonadotropic hypogonadism | 1277 | Congenital hypogonadotropic hypogonadism (174590) |
| Osteogenesis imperfecta | 1187 | Osteogenesis imperfecta (666) |
| Spinal muscular atrophy | 1154 | Proximal spinal muscular atrophy (70) |
| Leber hereditary optic neuropathy | 1153 | Leber hereditary optic neuropathy (104) |
| SAPHO syndrome | 990 | SAPHO syndrome (793) |
| Neurofibromatosis | 966 | Neurofibromatosis type 1 (636), Neurofibromatosis type 2 (637), Neurofibromatosis type 6 (2678), Neurofibromatosis type 3 (93921) |
| Lymphangioleiomyomatosis | 872 | Lymphangioleiomyomatosis (538) |
| Young-onset Parkinson disease | 758 | Young-onset Parkinson disease (2828) |
| Neuromyelitis Optica Spectrum Disorder | 754 | Neuromyelitis optica spectrum disorder (71211) |
| Amyotrophic lateral sclerosis | 668 | Amyotrophic lateral sclerosis (803) |
| Paroxysmal nocturnal hemoglobinuria | 618 | Paroxysmal nocturnal hemoglobinuria (447) |
| Idiopathic or cryptogenic familial epilepsy syndrome with identified loci/genes | 555 | Idiopathic or cryptogenic familial epilepsy syndrome with identified loci/genes (166475) |
| Light-chain amyloidosis | 534 | AL amyloidosis (85443) |
| POEMS syndrome | 532 | POEMS syndrome (2905) |
| Pulmonary alveolar proteinosis | 492 | Autoimmune pulmonary alveolar proteinosis (747) |
| Nevus Of Ota | 453 | Nevus of Ota (263425) |
| IgG4-related disease | 420 | IgG4-related disease (284264) |
| Systemic sclerosis | 393 | Systemic sclerosis (90291) |
| Hereditary spastic paraplegia | 318 | Hereditary spastic paraplegia (685) |
| Fabry disease | 298 | Fabry disease (324) |
| Autosomal recessive cerebellar ataxia | 287 | Autosomal recessive cerebellar ataxia (1172) |
| Charcot-Marie-Tooth disease | 279 | Charcot-Marie-Tooth disease/Hereditary motor and sensory neuropathy (166) |
| Gastrointestinal stromal tumor | 272 | Gastrointestinal stromal tumor (44890) |
| Moyamoya disease | 271 | Moyamoya angiopathy (477768) |
| Neuroendocrine carcinoma of pancreas | 251 | Neuroendocrine carcinoma of pancreas (506098) |
| Hepatolenticular degeneration | 245 | Wilson disease (905) |
| Retinitis pigmentosa | 241 | Retinitis pigmentosa (791) |
| Glycogen storage disease (Type Ia) | 240 | Glycogen storage disease due to glucose-6-phosphatase deficiency type Ia (79258) |
| Rare acquired aplastic anemia | 227 | Rare acquired aplastic anemia (164823) |
| Familial dilated cardiomyopathy | 226 | Familial dilated cardiomyopathy (217607) |
| Castleman disease | 221 | Castleman disease (160) |
| Eosinophilic granulomatosis with polyangiitis | 209 | Eosinophilic granulomatosis with polyangiitis (183) |
| Multiple endocrine neoplasia Type 2 | 186 | Multiple endocrine neoplasia type 2 (653) |
| Pelizaeus-merzbacher disease | 177 | Pelizaeus-Merzbacher disease (702) |
| Congenital coronary artery aneurysm | 157 | Congenital coronary artery aneurysm (95491) |
| Congenital uterine/genital tract malformation | 148 | Pseudounicornuate uterus (180079), True unicornuate uterus (180074), Unilateral aplasia of the Müllerian ducts (180071), Vaginal atresia (65681), Transverse vaginal septum (180160), Septate vagina (180154) |
| Hereditary peripheral neuropathy | 143 | Rare hereditary disease with peripheral neuropathy (207015) |
| Autoinflammatory disease | 132 | Autoinflammatory syndrome (93665) |
| Mucopolysaccharidosis Type I | 132 | Mucopolysaccharidosis type 1 (579) |
| Turner Syndrome | 127 | Turner syndrome (881) |
| Multiple endocrine neoplasia Type I | 119 | Multiple endocrine neoplasia type 1 (652) |
| Corneal dystrophy | 118 | Corneal dystrophy (34533) |
| Congenital long QT syndrome | 114 | Congenital long QT syndrome (768) |
| Familial isolated restrictive cardiomyopathy | 112 | Familial isolated restrictive cardiomyopathy (75249) |
| Rectal Neuroendocrine Neoplasms | 105 | Gastroenteric neuroendocrine neoplasm (481508) |
| 46,XY disorder of sex development | 100 | 46,XY disorder of sex development (98085) |
| Gaucher disease | 85 | Gaucher disease (355) |
| Cystic fibrosis | 85 | Cystic fibrosis (586) |
| Primary cutaneous lymphoma | 85 | Primary cutaneous lymphoma (542) |
| Left ventricular noncompaction | 80 | Left ventricular noncompaction (54260) |
| Mucopolysaccharidosis Type I | 80 | Mucopolysaccharidosis Type I (579) |
| Hypomyelinating Leukodystrophy | 68 | C11ORF73-related autosomal recessive hypomyelinating leukodystrophy (495844), NKX6-2-related autosomal recessive hypomyelinating leukodystrophy (527497), RARS-related autosomal recessive hypomyelinating leukodystrophy (438114), VPS11-related autosomal recessive hypomyelinating leukodystrophy (466934) |
| Pheochromocytoma-paraganglioma | 61 | Pheochromocytoma-paraganglioma (573163) |
| Alexander disease | 60 | Alexander disease (58) |
| Familial progressive hyperpigmentation | 54 | Familial progressive hyperpigmentation (79146) |
| CACH syndrome | 53 | CACH syndrome (135) |
| Primary ciliary dyskinesia | 53 | Primary ciliary dyskinesia (244) |
| Laron syndrome | 52 | Laron syndrome (633) |
| Inherited epidermolysis bullosa | 52 | Inherited epidermolysis bullosa (79361) |
| Gastric Neuroendocrine Neoplasm | 49 | Gastroenteric neuroendocrine neoplasm (481508) |
| Porphyria | 43 | Porphyria (738) |
| Treacher-Collins syndrome and Goldenhar syndrome | 38 | Treacher-Collins syndrome (861), Goldenhar syndrome (374) |
| Cronkhite-Canada syndrome | 37 | Cronkhite-Canada syndrome (2930) |
| Parathyroid carcinoma | 37 | Parathyroid carcinoma (143) |
| Megalencephalic leukoencephalopathy with subcortical cysts | 36 | Megalencephalic leukoencephalopathy with subcortical cysts (2478) |
| Reticulate acropigmentation of Kitamura | 36 | Reticulate acropigmentation of Kitamura (178307) |
| Peripartum cardiomyopathy | 36 | Peripartum cardiomyopathy (563) |
| Hereditary ATTR amyloidosis | 32 | Hereditary ATTR amyloidosis (271861) |
| Metachromatic leukodystrophy | 32 | Metachromatic leukodystrophy (512) |
| Generalized resistance to thyroid hormone | 31 | Generalized resistance to thyroid hormone (3221) |
| Isolated Klippel-Feil syndrome | 30 | Isolated Klippel-Feil syndrome (2345) |
| Prader-Willi syndrome | 30 | Prader-Willi syndrome (739) |
| maple syrup urine disease | 30 | maple syrup urine disease (511) |
| Congenital factor V deficiency | 28 | Congenital factor V deficiency (326) |
| Familial primary localized cutaneous amyloidosis | 28 | Familial primary localized cutaneous amyloidosis (353220) |
| Cerebral autosomal dominant arteriopathy-subcortical infarcts-leukoencephalopathy | 27 | Cerebral autosomal dominant arteriopathy-subcortical infarcts-leukoencephalopathy (136) |
| Globoid cell leukodystrophy (Krabbe disease) | 27 | Krabbe disease (487) |
| Porokeratosis | 26 | Porokeratosis (79358) |
| X-linked adrenoleukodystrophy | 24 | X-linked adrenoleukodystrophy (43) |
| Peutz-Jeghers syndrome | 24 | Peutz-Jeghers syndrome (2869) |
| Erythroderma desquamativum | 24 | Erythroderma desquamativum (314) |
| Langerhans cell histiocytosis | 23 | Langerhans cell histiocytosis (389) |
| Familial melanoma | 22 | Familial melanoma (618) |
| Arrhythmogenic right ventricular cardiomyopathy | 22 | Arrhythmogenic right ventricular cardiomyopathy (247) |
| Pyoderma gangrenosum | 21 | Pyoderma gangrenosum (48104) |
| Hereditary angioedema | 20 | hereditary angioedema (91378) |
| Brugada Syndrome | 19 | Brugada Syndrome (130) |
| Keratosis follicularis | 19 | Darier disease (218) |
| Familial generalized lentiginosis | 18 | Familial generalized lentiginosis (231040) |
| Epidermal nevus syndrome | 17 | Epidermal nevus syndrome (35125) |
| Incontinentia pigmenti | 17 | Incontinentia pigmenti (464) |
| Congenital factor VII deficiency | 17 | Congenital factor VII deficiency (327) |
| Homozygous familial hypercholesterolemia | 16 | Homozygous familial hypercholesterolemia (391665) |
| Blue rubber bleb nevus | 15 | Blue rubber bleb nevus (1059) |
| Autosomal dominant tubulointerstitial kidney disease | 15 | Autosomal dominant tubulointerstitial kidney disease (34149) |
| Pelizaeus-Merzbacher-like disease | 14 | Pelizaeus-Merzbacher-like disease (280270) |
| Ichthyosis | 14 | Ichthyosis (79354) |
| Autosomal dominant isolated diffuse palmoplantar keratoderma | 12 | Autosomal dominant isolated diffuse palmoplantar keratoderma (98349) |
| Chronic enteropathy associated with SLCO2A1 gene | 11 | Chronic enteropathy associated with SLCO2A1 gene (468641) |
| Congenital factor X deficiency | 10 | Congenital factor X deficiency (328) |
| Generalized pustular psoriasis | 9 | Generalized pustular psoriasis (247353) |
| Genetic renal or urinary tract malformation | 9 | Genetic renal or urinary tract malformation (183539) |
| Piebaldism | 7 | Piebaldism (2884) |
| Canavan disease | 6 | Canavan disease (141) |
| Sotos syndrome | 6 | Sotos syndrome (821) |
| Congenital factor II deficiency | 6 | Congenital factor II deficiency (325) |
| Ectodermal dysplasia syndrome | 4 | Ectodermal dysplasia syndrome (79373) |
| Angelman syndrome | 3 | Angelman syndrome (72) |
| Oculocutaneous albinism | 3 | Oculocutaneous albinism (55) |
| Non-amyloid monoclonal immunoglobulin deposition disease | 3 | Non-amyloid monoclonal immunoglobulin deposition disease (86861) |
| Tuberous sclerosis complex | 3 | Tuberous sclerosis complex (805) |
| Erythrokeratodermia variabilis | 3 | Erythrokeratodermia variabilis (317) |
| Dowling-Degos disease | 3 | Dowling-Degos disease (79145) |
| Xeroderma pigmentosum | 3 | Xeroderma pigmentosum (910) |
| Mitochondrial neurogastrointestinal encephalomyopathy | 3 | Mitochondrial neurogastrointestinal encephalomyopathy (298) |
| Marfan syndrome | 2 | Marfan syndrome (558) |
| Glycogen storage disease type II | 2 | Glycogen storage disease type II (365) |
| Pachyonychia congenita | 2 | Pachyonychia congenita (2309) |
| Livedoid vasculopathy | 1 | Livedoid vasculopathy (542643) |
| Epidermodysplasia verruciformis | 1 | Epidermodysplasia verruciformis (302) |
| Fragile X syndrome | 1 | Fragile X syndrome (908) |
| Familial multiple lentigines syndrome | 1 | Familial multiple lentigines syndrome (231040) |
| Fanconi anemia | 1 | Fanconi anemia (84) |
| Familial benign chronic pemphigus | 1 | Familial benign chronic pemphigus (2841) |
| Cat eye syndrome | 1 | Cat eye syndrome (195) |
| Dermatopathia pigmentosa reticularis | 1 | Dermatopathia pigmentosa reticularis (86920) |
| Systemic mastocytosis | 1 | Systemic mastocytosis (2467) |
| Dyskeratosis congenita | 1 | Dyskeratosis congenita (1775) |
| Non-amyloid fibrillary glomerulopathy | 1 | Non-amyloid fibrillary glomerulopathy (97566) |
| Small intestine Neuroendocrine Neoplasms | 1 | Small intestine Neuroendocrine Neoplasms (423975) |
| Vogt-Koyanagi-Harada disease | 1 | Vogt-Koyanagi-Harada disease (3437) |
| Nevus of ito | 1 | Nevus of ito (263432) |
| Hypotrichosis simplex | 1 | Hypotrichosis simplex (55654) |

Diseases with explicit ORPHAcode in NRDRS are presented
